# Malodors as environmental injustice: health symptoms in the aftermath of a hydrogen sulfide emergency in Carson, California, USA

**DOI:** 10.1038/s41370-023-00561-x

**Published:** 2023-06-30

**Authors:** Arbor J. L. Quist, Jill E. Johnston

**Affiliations:** Department of Population and Public Health Sciences, Keck School of Medicine, University of Southern California, 1845 N Soto St., Los Angeles, CA 90032 USA

**Keywords:** Air quality, Hydrogen sulfide, Environmental justice, Community-engaged research, Malodors; emergencies

## Abstract

In October 2021, hydrogen sulfide (H_2_S), a toxic odorous gas, was measured in Carson, California at concentrations reaching 7000 parts per billion (ppb), exceeding California’s 30 ppb acute air quality standard. Thousands of residents complained of malodors and headaches. We responded to community concerns by launching a rapid survey assessing symptoms. We recruited participants through door-to-door outreach, community events, and social media. During the emergency’s first week, 75% of the 108 total respondents experienced headaches, 72% experienced dizziness, and 63% experienced difficulty sleeping. About 60% of respondents (*n* = 63, no response = 7) noted the odor worsening their mental health. When adjusting for age, sex, and smoking, participants <2 km from the highest H_2_S concentrations reported higher prevalence of agitation (PD = 0.23, 95% CI: 0.03, 0.42) and headaches (PR = 0.14, 95% CI: −0.04, 1.31) compared to participants farther away. Malodors are underprioritized in environmental justice communities, despite H_2_S’s effects on mental and physical health.

## Introduction

Hydrogen sulfide (H_2_S) is a colorless, odorous gas characterized by a ‘rotten egg’ stench. H_2_S is released naturally into the environment by sulfur springs, volcanic gases, and decaying matter, and as a byproduct from industries such as industrial livestock, paper manufacturing, and oil and gas production [1, 2]. Many H_2_S-emitting industries are disproportionately located in low-income areas and in communities of color [3–5]. Exposure to H_2_S can cause severe acute symptoms—including unconsciousness and death at levels above 500 parts per million (ppm) [6]. At lower exposures—between 1 and 500 parts per billion (ppb)—documented acute impacts include headaches, throat irritation, watery eyes, shortness of breath, balance problems, and fatigue [1]. Malodors like H_2_S are environmental stressors, even at non-toxic levels; lack of control over one’s odor exposure can produce discomfort, fear, and anxiety in many people, with some experiencing extreme stress and stress-related symptoms [7–9].

In early October 2021, thousands of residents in Carson, California began complaining of noxious odor, headaches, breathing problems, and nausea [10]. The local regulatory agency, the South Coast Air Quality Management District (SCAQMD), received over 4000 odor complaints within 1 week from residents regarding a noxious rotten-egg-like smell [11]. Initial investigations measured hydrogen sulfide (H_2_S) levels of 400–900 parts per billion (ppb) near the Dominguez Channel—a 25 km flood-control concrete waterway in the densely populated area of the South Bay in Los Angeles (LA) County. The levels continued to increase and peaked in mid-October at around 7000 ppb—230 times the California’s acute ambient air quality standard of 30 ppb [12, 13]. Strong malodors and adverse health effects associated with them plagued the predominantly low-income communities of color in Carson and the South Bay for over 2 months [13, 14].

The cause of the H_2_S spike in Carson was unknown for 2 months, during which time government officials attributed the H_2_S to the natural decay of organic materials in the Channel [15]. In December 2021, SCAQMD issued notices of violation to four companies and the County of LA [11]. According to their investigation, the H_2_S release was connected to a large warehouse fire on September 30, 2021 in the City of Carson that resulted in chemicals, including ethanol, benzene, and isopropyl alcohol, being flushed into the Dominguez Channel. They concluded that this facilitated the anaerobic decay of organic materials in the Channel which subsequently resulted in the release massive quantities of H_2_S. Environmental odors were not new to the Carson community; in 2018–2020, about 60% of environmental complaints placed to SCAQMD by Carson area residents were odor complaints, but this rose to 93% in 2021 [16]. Many oil refineries, industrial facilities, and freeways have been concentrated in Carson for decades. Carson is ranked in the top quartile of statewide pollution burden and population vulnerability according to the California Communities Environmental Health Screening Tool, with an especially high pollution burden of toxic releases, fine particulate matter, and hazardous waste [17]. The H_2_S emergency in the fall of 2021 exacerbated existing environmental justice issues in Carson.

In this paper, we describe a rapid response to record health symptoms through a survey of residents in the Carson area to understand health impacts of this malodorous event [10, 18]. While there is a body of literature surrounding the acute impacts of H_2_S exposures, health effects in the aftermath of a malodorous emergency in an urban environmental justice community like this are not well characterized.

## Methods

We recruited participants using a combination of outreach to community-based organizations, as well as through the use of direct mailing, door-to-door outreach, and social media in targeted areas. As a result of a longstanding partnership with the community-based organization the Coalition for a Safe Environment, we were approached to provide information about the health impacts of H_2_S exposure and to speak with residents and public officials, both during and after the odor incident. We used these opportunities to recruit participants for the health survey. Volunteers with a local nonprofit telephoned hundreds of Carson residents to create a database of concerned citizens and to recruit residents for this health survey. Study eligibility included: at least 18 years of age, fluency in English or Spanish, and residing in the City of Carson, or adjacent neighborhoods of West Carson, Gardena, Torrance, Wilmington, or Long Beach since August 2021. This study was approved by the University of Southern California Institutional Review Board. Informed consent was obtained from each participant before they completed the survey.

We analyzed H_2_S concentrations at SCAQMD monitors during the emergency and calculated the number of hours above the state’s 1 h acute standard of 30 ppb. We calculated the distance from each participant’s address to the Dominguez Channel in Carson and categorized participants living <2 km from the Channel in Carson as living near high H_2_S exposure and participants living ≥2 km from the Channel as farther from high H_2_S exposure. We calculated the prevalence differences of symptoms experienced during the worst week of the event by residents living near the Channel in Carson compared to residents living farther from the Channel using a generalized linear model with a Gaussian distribution [19]. We used the robust variance estimator to calculate 95% confidence intervals. We adjusted for sex, age category (18–44, 45–64, 65 + ), and current smoking status. We also stratified analyses by asthma and allergy status. R version 4.1.0 was used for all analyses [20].

## Results

**Fig. 1:**
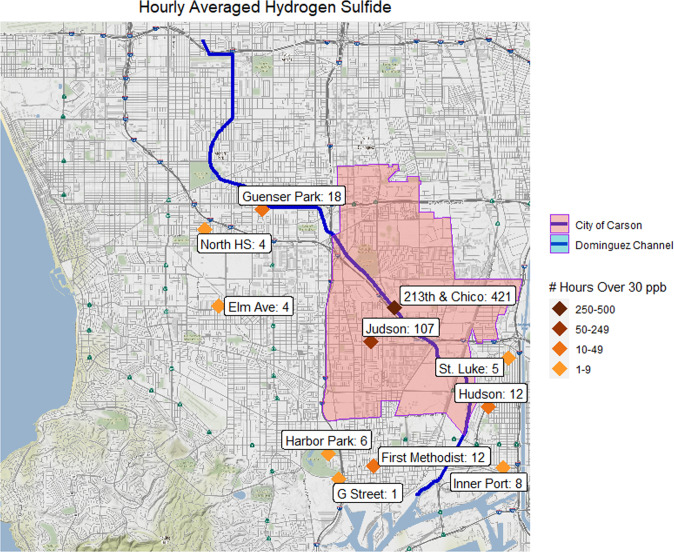
Frequency of hydrogen sulfide exceeding California’s acute standard. Map of stationary hydrogen sulfide monitors in South Bay of Los Angeles and the number of hours the monitors measured hourly average H_2_S concentration above 30 ppb from October 1, 2021 to January 31, 2022.

Based on analysis of H_2_S monitors in Carson and surrounding areas, the hourly H_2_S averages were above 30 ppb for 490 h between October 1, 2021 and January 31, 2022 (Fig. 1). From November 2021 to May 2022, 109 participants completed the health survey. One participant was excluded because they only completed the odor section of the survey, leaving a study sample of 108 participants. All participants who completed the odor and health sections of the survey were included in descriptive analyses (*N* = 108), although five of these participants did not provide demographic information and two participants did not provide their residential address (*N* = 106 for distance-related analyses). An estimated 52% of the participants (*n* = 56, no response = 2) live <2 km from Dominguez Channel in Carson and 46% reside farther from the Channel (2–8 km from Channel, *n* = 50). The median distance from participant house to the Dominguez Channel in Carson was 1.8 km, with a range from 130 m to 7.8 m. About 65% of participants were women (Table 1, *n* = 70, no response = 6). Participants ranged in age from 18 to 96 (median = 42, no response = 6). About a third of the participants identified as Hispanic/Latinx (*n* = 37, no response = 5), with 34% identifying as white (*n* = 34, no response = 5), 22% as Black (*n* = 24), and 15% as Asian (*n* = 16). While the majority of participants completed the survey online by an individualized link we emailed to each interested participant, almost 20% completed the survey in person or by phone with a research assistant. Six participants completed the survey in Spanish. Half of the 108 participants completed the survey in November and December 2021, and 92% of participants completed the survey before the end of March 2022.

**Table 1:** Characteristics of survey respondents.

Characteristic	Number of Respondents (Percent)
*Sex*	
Female	70 (65)
Male	31 (29)
Nonbinary	1 (1)
No response	6 (6)
*Race/ethnicity*	
Hispanic/Latinx	37 (34)
Asian	16 (15)
Black	24 (22)
Native American	6 (6)
Native Hawaiian/Pacific Islander	5 (5)
White	34 (31)
Another race	18 (17)
No response	5 (5)
*Housing Type*	
Single family house	57 (53)
Duplex/quadruplex	11 (10)
Apartment	9 (8)
Mobile home park/manufactured home	23 (21)
Other	3 (3)
No response	5 (5)
*Housing status*	
Rent home	25 (23)
Own home	51 (47)
Own home, rent space (mobile home park)	22 (20)
Other	5 (5)
No response	5 (5)
*Health insurance*	
Medi-Cal	36 (33)
My Health LA	1 (1)
Covered California Plan	6 (6)
Private health insurance	55 (51)
No health insurance	6 (6)
No response	4 (4)
*Highest completed formal education*	
Less than high school degree	5 (5)
High school graduate	14 (13)
Some university/college	31 (29)
Technical or trade school	7 (6)
Junior/community college	9 (8)
Bachelor’s degree	26 (24)
Graduate degree	13 (12)
No response	5 (5)
*Employment status*	
Employed	60 (56)
Unemployed – seeking work	12 (11)
Unemployed – retired	22 (20)
Unemployed – homemaker	4 (4)
Unemployed – disabled	1 (1)
Student	3 (3)
No response	5 (5)
Current smoker^a^	15 (14)
Years in current home (median, IQR)^a^	10, 21
Age (median, IQR)^b^	42, 32

^a^8 non-responses.

^b^6 non-responses.

Eighty-four percent of participants (91 of 108 respondents) smelled new strong odors in October 2021 and 31% of participants (*n* = 34, no response = 2) relocated from their home during the emergency. A third of surveyed residents (*n* = 37, no response = 3) reported receiving a medical evaluation because of odor-related symptoms. Three-fourths of participants (*n* = 81, no response = 9) reported that odors caused them to decrease their time outside and to open the window less. Only 13% of participants (*n* = 14, no response = 1) felt that the odor issue had been completely resolved at the time of the survey, with 45% indicating it has been partly resolved. Half of the respondents who indicated that the odor incident had been resolved completed the survey in November and December 2021 during the end of the event. Several respondents noted that the odor has been resolved but the health effects that started with the odor event remained. During the first week of the malodor event, 75% of the 108 respondents reported headaches, 72% reported dizziness, 66% reported burning eyes and fatigue, 65% reported nausea, and 63% reported difficulty sleeping (Table 2). About half of participants reported anxiety, difficulty concentrating, and difficulty breathing during the event. In the 1–4 months after the event, a third of participants continued to report fatigue, headache, and burning eyes; 30% (*n* = 32) continued to report dizziness and 24% (*n* = 26) continued to experience anxiety and burning throat. Prevalence of reported symptoms was similar among participants surveyed during vs. after the emergency (Supplementary Table 1). Almost seventy percent of respondents (*n* = 73, no response = 4) reported that the odor worsened their physical health and 58% (*n* = 63, no response = 7) reported that it worsened their mental health. Doctor-diagnosed asthma was reported by 28% of participants (*n* = 30, no response = 1), and of those people, 63% (19 of 30) reported that their asthma was worse during the odor event. Almost half of respondents reported having doctor-diagnosed allergies (*n* = 49, no response = 5), with 69% (34 of 49) of these people saying their allergies were worse during the event.

**Table 2:** Prevalence difference (and 95% confidence interval) of symptoms experienced during the first week of the event in residents <2 km from Dominguez Channel compared to residents living ≥2 km from Channel, adjusting for age category, sex, and current smoking status.

	Symptoms	Adjusted prevalence difference (95% CI)	Number of participants reporting symptom
Neurological	Headache	0.14 (−0.04, 0.31)	81
	Dizziness	−0.02 (−0.20, 0.15)	78
	Nausea	0.05 (−0.15, 0.24)	70
	Difficulty concentrating	−0.04 (−0.25, 0.16)	52
	Numbness	−0.1 (−0.30, 0.09)	36
	Loss of balance	0.04 (−0.14, 0.22)	33
	Memory loss	0.14 (−0.04, 0.32)	27
	Blurred vision	−0.09 (−0.29, 0.11)	45
Respiratory	Burning eyes	−0.15 (−0.34, 0.04)	72
	Runny nose	−0.22 (−0.41, −0.04)	68
	Burning nose/throat	−0.03 (−0.22, 0.16)	66
	Cough	0.05 (−0.15, 0.25)	64
	Difficulty breathing	0.17 (−0.04, 0.37)	52
	Increased congestion	−0.01 (−0.21, 0.20)	52
	Tearing eyes	−0.02 (−0.22, 0.19)	49
	Burning lungs	0.04 (−0.14, 0.22)	33
	Nosebleeds	−0.02 (−0.16, 0.13)	19
	Difficulty swallowing	−0.16 (−0.36, 0.03)	38
	Wheezing	−0.01 (−0.21, 0.18)	38
	Chest pain	0.07 (−0.12, 0.26)	30
Well-being	Fatigue	0.15 (−0.04, 0.34)	71
	Difficulty sleeping	0.02 (−0.18, 0.22)	68
	Anxiety	0.11 (−0.09, 0.31)	53
	Agitation	0.23 (0.03, 0.42)	50
	Depression	−0.03 (−0.23, 0.18)	48
	General weakness	0.02 (−0.17, 0.22)	35
Gastrointestinal	Abdominal pain	0.13 (−0.05, 0.30)	28
	Diarrhea	0.10 (−0.05, 0.25)	22
	Vomiting	−0.08 (−0.23, 0.07)	18
Muscular	Arm weakness	0.06 (−0.09, 0.22)	20
	Leg weakness	0.09 (−0.06, 0.24)	21
	Muscle twitching	0.09 (−0.08, 0.26)	21
	Arm/leg tremors	0.08 (−0.07, 0.23)	18

Participants living within 2 km from the Dominguez Channel in Carson reported higher prevalence of headaches (prevalence difference [PD] = 0.14, 95% confidence interval [CI]: −0.04, 0.31), difficulty breathing (PD = 0.17, 95% CI: −0.04, 0.37), fatigue (PD = 0.15, 95% CI: −0.04, 0.34), and agitation (PD = 0.23, 95% CI: 0.03, 0.42), and a lower prevalence of runny nose (PD = −0.22, 95% CI: −0.41, −0.04), compared to participants ≥2 km from Channel. The prevalence of the other commonly reported symptoms, including dizziness, nausea, burning nose/throat, difficulty sleeping, and depression, was similar among those who live <2 km and 2–8 km from Dominguez Channel during the odor event (Table 2). When restricted to participants with doctor-diagnosed asthma, albeit only 30 participants, there was a higher prevalence of agitation (PD = 0.46, 95% CI: 0.15, 0.77, Supplementary Table 2) and fatigue (PD = 0.43, 95% CI: 0.02, 0.67) among participants living near the high H_2_S exposure compared to those living farther from the exposure. Similar results were seen when restricted to participants with doctor-diagnosed allergies (*N* = 49; agitation PD = 0.42, 95% CI: 0.16, 0.68; fatigue PD = 0.26, 95% CI:−0.01, 0.53).

## Discussion

Headaches, dizziness, fatigue, burning eyes, runny nose, nausea, and difficulty sleeping were the most common symptoms reported during the immediate aftermath of the hydrogen sulfide event. Participants living near the Dominguez Channel in Carson—where higher levels of H_2_S was measured—reported a higher prevalence of agitation, difficulty breathing, headaches, and fatigue. The prevalence of the remaining reported health symptoms, including dizziness, burning and tearing eyes, coughing, and difficulty sleeping, were similar in residents living near and farther from the Channel, highlighting that these symptoms related to the H_2_S event impacted residents in a large geographic region.

The most frequently reported symptoms have been common symptoms in other studies of H_2_S exposure. A community-based study found higher rates of self-reported fatigue, headache, dizziness, anxiety, shortness of breath, and difficulty sleeping in residents of communities with chronic low-level H_2_S exposure (annual averages ranging from 1 to 27 ppb, with occasional spikes of 500 ppb) compared to unexposed residents of reference communities [21]. Another study observed a higher prevalence of coughing, nausea, and eye irritation during a high H_2_S exposure period (daily averages: 24–30 ppb, max: 94 ppb) in Finnish community near a pulp mill compared to a low-exposure period (daily averages 0.07–2.4 ppb) [22]. Increasing concentrations of H_2_S has been associated with increases in irritation and anxiety [23], and almost half of our study participants reported anxiety and agitation with a higher prevalence of agitation among residents in higher exposed areas. Even low H_2_S concentrations (1–90 ppb) have been associated with stress and negative mood [24], as these low levels are often experienced by many humans as strong malodors. Most individuals are able to detect H_2_S odor at levels ~0.1–0.5 ppb [25]. The US EPA has a reference concentration for chronic inhalation exposure of 1.4 ppb [26], which indicates the estimated level of daily H_2_S concentration that humans (including sensitive populations) can be exposed to without significant risk of adverse health effects. For episodic exposure, the state of CA 1 h average ambient air quality standard is 30 ppb, which has been unchanged since adopted in 1969 [12].

Sensitive populations may have been especially affected during this emergency. Very few studies have examined how H_2_S exposure affects vulnerable populations, including people with asthma or other respiratory problems. Bates et al. assessed lung function among 1204 residents living with chronic low-level H_2_S (median: 20.3 ppb, range 0–64 ppb) in New Zealand and did not observe long-term H_2_S exposure to be associated with reduced lung function, even among participants with asthma or COPD [27]. Our results do not confirm their findings, as many surveyed participants with existing respiratory conditions reported worsened symptoms during the H_2_S event. A study using the Chemical Odor Sensitivity Scale found that people with asthma and allergies are more sensitive to chemical odors than those without these respiratory issues [28]. Chronic H_2_S exposure may affect sensitive populations differently than acute H_2_S exposures, which may explain why previous studies of chronic low-level H_2_S exposure have not been associated with reduced lung function among those with existing respiratory conditions. In addition, age, perceived health status, stress coping styles, and other individual-level factors have been found to modify odor responses [9]. Humans experience odors differently, and more research is needed to understand how H_2_S exposure affects sensitive and vulnerable populations during H_2_S emergencies and to establish enforceable regulatory standards for H_2_S that consider sensitive populations.

The study’s strengths include its rapid launch of a community survey during a H_2_S emergency (and a pandemic). We recruited participants using diverse methods and were able to recruit a sample with similar demographics to that of the City of Carson. However, the study is limited by its small sample size and lack of a clear control group. While we recruited participants who lived 130 m–8 km from the Dominguez Channel in Carson, the majority of participants smelled the odors and were affected in some way by the incident. While some of our recruitment methods may have increased selection bias by recruiting participants who were especially concerned about or affected by the odors, when we went door to door, we were able to enroll Carson residents who did not smell the H_2_S or were not substantially bothered by the odors. As with all surveys, this study is limited by the self-report nature of the data and potential recall biases of the participants. The degree of recall bias may change over time, with participants surveyed during the incident experiencing less recall bias than the participants we surveyed several months after the incident. However, the majority of respondents completed the survey in the first 3 months of the event in November 2021–January 2021.

## Conclusions

H_2_S exposure is an environmental justice issue, as communities exposed to H_2_S and other malodors are commonly communities of color and low-income communities [24, 29–31]. Malodors are typically overlooked and underprioritized in these environmental justice communities while they continue to harm residents. According to this community survey, the Dominguez Channel H_2_S incident affected the health of nearby residents and was associated with headaches, difficulty breathing, fatigue, and agitation. Our findings suggest that reducing H_2_S exposure by regulating it as a toxic air pollutant at lower concentrations may improve the mental and physical health of exposed residents.

## Supplementary information

Supplementary tables

## Data Availability

The survey data that support the findings of this study are not publicly available to safeguard the privacy of the participants and to maintain trust with affected communities. Data may be available from the authors upon reasonable request and with permission of the University of Southern California Institutional Review Board. Hydrogen sulfide data is available through South Coast Air Quality Management District at https://xappprod.aqmd.gov/Rule1180CommunityAirMonitoring/
