# Supplementary material for: Malodors as environmental injustice: health symptoms in the aftermath of a hydrogen sulfide emergency in Carson, California, USA
Source: J Expo Sci Environ Epidemiol. 2023 Jun 30;34(6):935–40. doi: 10.1038/s41370-023-00561-x (PMC10792538; doi:10.1038/s41370-023-00561-x)
Supplement: Supplementary file 1 — Supplementary tables [file 41370_2023_561_MOESM1_ESM.docx]

**Supplementary Table 1. Prevalence of symptoms experienced during the first week of the event among all participants, stratified by time period when participant completed survey (Pearson’s chi-squared test p-value shown to compare groups). None of the reported symptoms are significantly different between time periods, although a larger proportion of participants surveyed during the event (Nov 2021-Jan 2022) reported blurred vision, agitation, difficulty swallowing, diarrhea, and vomiting. A larger proportion of participants surveyed after the event (Feb-Apr 2022) reported muscle twitching and burning eyes.**

| Symptoms | Number of participants reporting symptom among those surveyed in Nov, Dec, Jan; N=62; n (%) | Number of participants reporting symptom among those surveyed in Feb, Mar, Apr; N=44; n (%) | p-value |
| --- | --- | --- | --- |
| Headache | 45 (72.6) | 35 (79.5) | 0.554 |
| Dizziness | 45 (72.6) | 33 (75.0) | 0.956 |
| Nausea | 39 (62.9) | 30 (68.2) | 0.723 |
| Difficulty concentrating | 33 (53.2) | 19 (43.2) | 0.411 |
| Numbness | 20 (32.3) | 16 (36.4) | 0.817 |
| Loss of balance | 17 (27.4) | 16 (36.4) | 0.443 |
| Memory loss | 17 (27.4) | 10 (22.7) | 0.749 |
| Blurred vision | 29 (46.8) | 16 (36.4) | 0.385 |
| Burning eyes | 38 (61.3) | 33 (75.0) | 0.204 |
| Runny nose | 41 (66.1) | 27 (61.4) | 0.765 |
| Burning nose/throat | 38 (61.3) | 28 (63.6) | 0.966 |
| Cough | 38 (61.3) | 26 (59.1) | 0.979 |
| Difficulty breathing | 29 (46.8) | 22 (50.0) | 0.896 |
| Increased congestion | 31 (50.0) | 21 (47.7) | 0.973 |
| Tearing eyes | 27 (43.5) | 22 (50.0) | 0.646 |
| Burning lungs | 20 (32.3) | 13 (29.5) | 0.933 |
| Nosebleeds | 10 (16.1) | 9 (20.5) | 0.753 |
| Difficulty swallowing | 25 (40.3) | 13 (29.5) | 0.35 |
| Wheezing | 23 (37.1) | 15 (34.1) | 0.91 |
| Chest pain | 17 (27.4) | 13 (29.5) | 0.984 |
| Fatigue | 41 (66.1) | 30 (68.2) | 0.991 |
| Difficulty sleeping | 37 (59.7) | 30 (68.2) | 0.49 |
| Anxiety | 33 (53.2) | 20 (45.5) | 0.554 |
| Agitation | 32 (51.6) | 18 (40.9) | 0.373 |
| Depression | 29 (46.8) | 19 (43.2) | 0.866 |
| General weakness | 20 (32.3) | 15 (34.1) | 1 |
| Abdominal pain | 17 (27.4) | 11 (25.0) | 0.956 |
| Diarrhea | 14 (22.6) | 5 (11.4) | 0.22 |
| Vomiting | 12 (19.4) | 4 (9.1) | 0.238 |
| Arm weakness | 9 (14.5) | 11 (25.0) | 0.268 |
| Leg weakness | 11 (17.7) | 10 (22.7) | 0.699 |
| Muscle twitching | 9 (14.5) | 12 (27.3) | 0.169 |
| Arm/leg tremors | 11 (17.7) | 7 (15.9) | 1 |

**Supplementary Table 2. Prevalence difference (and 95% confidence intervals) of symptoms experienced during the first week of the event in residents <2 km from Dominguez Channel compared to residents living ≥2 km from Channel, adjusting for age category, sex, and current smoking status, stratified by doctor-diagnosed allergy status and asthma status. Please note small sample size for these analyses. Among participants with doctor-diagnosed asthma, albeit only 30 participants, there was a higher prevalence of agitation and fatigue among participants living near the high hydrogen sulfide exposure compared to those living farther away. Similar results for agitation and fatigue were seen when restricted to participants with doctor-diagnosed allergies (N=49).**

| Symptoms | Prevalence difference among participants with allergies (N=49) | Prevalence difference among participants without allergies (N=49) | Prevalence difference among participants with asthma (N=30) | Prevalence difference among participants without asthma (N=70) |
| --- | --- | --- | --- | --- |
| Headache | 0.12 (-0.14, 0.37) | 0.1 (-0.12, 0.33) | 0.1 (-0.26, 0.46) | 0.19 (-0.01, 0.4) |
| Dizziness | -0.01 (-0.27, 0.25) | -0.1 (-0.38, 0.19) | -0.02 (-0.32, 0.28) | 0.06 (-0.18, 0.3) |
| Nausea | -0.12 (-0.38, 0.15) | 0.15 (-0.1, 0.41) | 0.17 (-0.19, 0.52) | 0.06 (-0.19, 0.31) |
| Difficulty concentrating | 0.04 (-0.27, 0.36) | -0.09 (-0.39, 0.21) | 0.27 (-0.03, 0.56) | -0.05 (-0.31, 0.2) |
| Numbness | -0.02 (-0.31, 0.27) | -0.3 (-0.6, 0.01) | -0.26 (-0.61, 0.08) | 0.04 (-0.21, 0.28) |
| Loss of balance | 0.08 (-0.18, 0.34) | -0.09 (-0.37, 0.18) | -0.12 (-0.42, 0.18) | 0.14 (-0.09, 0.38) |
| Memory loss | 0.16 (-0.1, 0.42) | 0.05 (-0.23, 0.34) | -0.01 (-0.29, 0.28) | 0.24 (0.01, 0.46) |
| Blurred vision | -0.02 (-0.32, 0.27) | -0.13 (-0.43, 0.16) | -0.24 (-0.57, 0.08) | 0.03 (-0.22, 0.27) |
| Burning eyes | -0.15 (-0.41, 0.12) | -0.12 (-0.43, 0.18) | -0.36 (-0.69, -0.03) | 0 (-0.25, 0.24) |
| Runny nose | -0.26 (-0.51, 0) | -0.16 (-0.47, 0.15) | -0.38 (-0.73, -0.03) | -0.18 (-0.41, 0.04) |
| Burning nose/throat | -0.17 (-0.44, 0.1) | 0.05 (-0.23, 0.33) | -0.28 (-0.64, 0.07) | 0.15 (-0.08, 0.37) |
| Cough | 0.21 (-0.06, 0.48) | -0.08 (-0.36, 0.21) | 0.07 (-0.32, 0.46) | 0.04 (-0.21, 0.29) |
| Difficulty breathing | 0.05 (-0.24, 0.35) | 0.24 (-0.05, 0.53) | 0.23 (-0.09, 0.55) | 0.13 (-0.11, 0.37) |
| Increased congestion | 0.05 (-0.23, 0.33) | -0.21 (-0.5, 0.08) | 0.13 (-0.23, 0.48) | -0.1 (-0.36, 0.16) |
| Tearing eyes | -0.12 (-0.43, 0.18) | 0.17 (-0.11, 0.44) | -0.36 (-0.7, -0.01) | 0.17 (-0.09, 0.44) |
| Burning lungs | 0.11 (-0.17, 0.38) | -0.09 (-0.33, 0.15) | -0.06 (-0.43, 0.31) | 0.12 (-0.1, 0.34) |
| Nosebleeds | 0.01 (-0.19, 0.2) | -0.06 (-0.3, 0.18) | 0.06 (-0.17, 0.28) | -0.03 (-0.23, 0.16) |
| Difficulty swallowing | -0.22 (-0.52, 0.07) | -0.24 (-0.48, -0.01) | -0.22 (-0.57, 0.12) | -0.16 (-0.41, 0.08) |
| Wheezing | 0.01 (-0.27, 0.3) | -0.06 (-0.33, 0.21) | -0.02 (-0.37, 0.33) | 0.03 (-0.2, 0.27) |
| Chest pain | 0 (-0.29, 0.28) | -0.03 (-0.31, 0.26) | -0.17 (-0.48, 0.13) | 0.21 (-0.02, 0.44) |
| Fatigue | 0.26 (-0.01, 0.53) | 0.03 (-0.25, 0.31) | 0.43 (0.20, 0.67) | 0.10 (-0.13, 0.34) |
| Difficulty sleeping | 0.06 (-0.23, 0.36) | -0.05 (-0.32, 0.21) | 0.1 (-0.22, 0.41) | 0.08 (-0.17, 0.32) |
| Anxiety | 0.05 (-0.24, 0.35) | 0.12 (-0.17, 0.41) | 0.08 (-0.29, 0.46) | 0.17 (-0.07, 0.42) |
| Agitation | 0.42 (0.16, 0.68) | 0.01 (-0.29, 0.3) | 0.46 (0.15, 0.77) | 0.12 (-0.13, 0.38) |
| Depression | -0.14 (-0.42, 0.14) | 0.03 (-0.28, 0.33) | -0.05 (-0.39, 0.3) | 0.02 (-0.25, 0.29) |
| General weakness | -0.01 (-0.29, 0.27) | -0.04 (-0.3, 0.23) | -0.08 (-0.4, 0.24) | 0.09 (-0.16, 0.33) |
| Abdominal pain | 0.17 (-0.09, 0.43) | 0.06 (-0.21, 0.33) | 0.07 (-0.24, 0.38) | 0.12 (-0.12, 0.35) |
| Diarrhea | 0.21 (-0.04, 0.47) | 0.02 (-0.17, 0.21) | 0.17 (-0.15, 0.48) | 0.08 (-0.07, 0.22) |
| Vomiting | 0.04 (-0.21, 0.3) | -0.19 (-0.39, 0.02) | 0.04 (-0.26, 0.33) | -0.12 (-0.29, 0.05) |
| Arm weakness | 0.05 (-0.19, 0.29) | -0.01 (-0.18, 0.16) | -0.01 (-0.31, 0.29) | 0.17 (0.01, 0.32) |
| Leg weakness | 0.02 (-0.24, 0.29) | 0.17 (0, 0.35) | -0.03 (-0.31, 0.25) | 0.21 (0.04, 0.37) |
| Muscle twitching | 0.32 (0.1, 0.54) | -0.18 (-0.44, 0.09) | 0.18 (-0.08, 0.44) | 0.11 (-0.1, 0.31) |
| Arm/leg tremors | 0.17 (-0.06, 0.41) | -0.11 (-0.33, 0.11) | 0.07 (-0.17, 0.31) | 0.14 (-0.04, 0.32) |
